# Whole-Genome Sequence of *Pseudomonas fluorescens* EK007-RG4, a Promising Biocontrol Agent against a Broad Range of Bacteria, Including the Fire Blight Bacterium* Erwinia amylovora*

**DOI:** 10.1128/genomeA.00026-17

**Published:** 2017-03-30

**Authors:** Roghayeh Habibi, Saeed Tarighi, Javad Behravan, Parissa Taheri, Annelise Helene Kjøller, Asker Brejnrod, Jonas Stenløkke Madsen, Søren Johannes Sørensen

**Affiliations:** a Section of Phytopathology, Department of Plant Protection, Ferdowsi University of Mashhad, Mashhad, Iran; b Section of Microbiology, Department of Biology, University of Copenhagen, Copenhagen, Denmark; c Section of Pharmaceutical Biotechnology, Department of Pharmacy, Mashhad University of Medical Sciences, Mashhad, Iran

## Abstract

Here, we report the first draft whole-genome sequence of *Pseudomonas fluorescens* strain EK007-RG4, which was isolated from the phylloplane of a pear tree. *P. fluorescens* EK007-RG4 displays strong antagonism against *Erwinia amylovora*, the causal agent for fire blight disease, in addition to several other pathogenic and non-pathogenic bacteria.

*Pseudomonas fluorescens* is a Gram negative, rod-shaped bacterium that is widely distributed in various environments (1, 2). The *P. fluorescens* group is one of the most diverse within the *Pseudomonas* genus, currently including more than 50 named species (3). Many strains belonging to the *P. fluorescens* group are plant commensals with efficient biocontrol properties (1, 3).

*P. fluorescens* EK007-RG4 was isolated from the phyllosphere of a pear tree in northern Iran. The strain produces compounds that are highly inhibitory against *Erwinia amylovora*, the common causal agent of fire blight disease, in addition to a diverse array of Gram-negative and -positive bacteria. The genome of EK007-RG4 was sequenced to further the understanding of the antibacterial properties of the strain.

Genomic DNA was extracted using the DNA blood and tissue kit from Qiagen. The whole-genome sequencing library was prepared using the Nextera XT DNA library preparation kit (Illumina) and quantified by a fragment analyzer (Advanced Analytical Technologies, Inc.). Sequencing was completed with 2 × 250-bp paired-end reads using the Illumina MiSeq platform (Illumina). Standard protocols were used for all of the above kits, as provided by the manufacturers. The reads were cleaned and trimmed (trim sequences tool; settings: ambiguous limit = 2, quality limit = 0.05) using CLC Genomics Workbench version 7 (CLC bio). Next, quality-filtered reads were assembled into contigs (*de novo* assembly tool; default settings, including both paired and orphaned reads; minimum contig length = 500 bp). The RAST and SEED (4) servers were used to annotate the resulting contigs.

The draft genome of *P. fluorescens* EK007-RG4 was 5,970,564 bp long, with an average coverage of 207×, and was assembled into 27 contigs with an average G+C content of 60.2% and 67 RNAs. RNAmmer analysis predicted 21 copies of the 5s and three copies each of the 23s and 16s rRNAs. The results of sequence annotation revealed 5,318 coding regions with 53% assigned to a SEED subsystem.

Genome comparison showed closest similarity between EK007-RG4 and the biocontrol agents *P. fluorescens* A506 and *P. fluorescens* SS101. Unique genes homologous to the nonribosomal peptide synthase genes *massA*, *massB*, and *massC* (5) were identified in the EK007-RG4 genome. These genes code for enzymes that biosynthesize cyclic lipopeptides (CLPs), which are biosurfactants with antimicrobial properties (6). A protein BLAST search of the translated *massA*,* massB*, and* massC* genes of EK007-RG4 showed 96%, 97%, and 97% similarity, respectively, to any GenBank sequences. The core structure of the putative *mass* CLP produced by EK007-RG4 was predicted as (lipid tail)-Leu-Asp-Thr-Ile-Leu-Ser-Leu-Ser-Ile using antiSMASH (7). This predicted CLP is different from any known nonribosomal peptides in the Norine database (8). The potentially new EK007-RG4 CLP is likely a type of viscosin. However, the putative EK007-RG4 CLP is unique in that the second amino acid is Asp, while it is consistently Glu among known viscosins.

## Accession number(s).

This whole-genome shotgun project has been deposited in DDBJ/ENA/GenBank under the accession number MRST00000000. The version described in this paper is the first version, MRST01000000.
